# Comparative immunogenicity of preparations of yeast-derived dengue oral vaccine candidate

**DOI:** 10.1186/s12934-018-0876-0

**Published:** 2018-02-16

**Authors:** Jyotiranjan Bal, Nguyen Ngoc Luong, Jisang Park, Ki-Duk Song, Yong-Suk Jang, Dae-Hyuk Kim

**Affiliations:** 1 Department of Molecular Biology, Department of Bioactive Material Sciences, Institute for Molecular Biology and Genetics, Chonbuk National University, Jeonju, Jeollabuk-do 54896 Republic of Korea; 2 Department of Biology, College of Sciences, Hue University, Hue, Vietnam; 3 Department of Animal Biotechnology, The Animal Molecular Genetics and Breeding Center, Chonbuk National University, Jeonju, Jeollabuk-do 54896 Republic of Korea

**Keywords:** Dengue, Oral vaccine, scEDIII, *Saccharomyces cerevisiae*, Neutralization, Mucosal immunity

## Abstract

**Background:**

Dengue is listed as a neglected tropical disease by the Center for Disease Control and Preservation, as there are insufficient integrated surveillance strategies, no effective treatment, and limited licensed vaccines. Consisting of four genetically distinct serotypes, dengue virus (DENV) causes serious life-threatening infections due to its complexity. Antibody-dependent enhancement by pre-existing cross-reactive as well as homotypic antibodies further worsens the clinical symptoms of dengue. Thus, a vaccine conferring simultaneous and durable immunity to each of the four DENV serotypes is essential to restrict its escalation. In deeply affected resource-limited countries, oral vaccination using food-grade organisms is considered to be a beneficial approach in terms of costs, patient comfort, and simple logistics for mass immunization. The current study used a mouse model to explore the immunogenicity of an oral dengue vaccine candidate prepared using whole recombinant yeast cells (WC) and cell-free extracts (CFE) from cells expressing recombinant *Escherichia coli* heat-labile toxin protein B-subunit (LTB) fused to the consensus dengue envelope domain III (scEDIII). Mice were treated orally with recombinant WC and CFE vaccines in 2-week intervals for 4 weeks and changes in systemic and mucosal immune responses were monitored.

**Results:**

Both WC and CFE dosage applications of LTB-scEDIII stimulated a systemic humoral immune response in the form of dengue-specific serum IgG as well as mucosal immune response in the form of secretory sIgA. Antigen-specific B cell responses in isolated lymphoid cells from the spleen and Peyer’s patches further indicated an elevated mucosal immune response. Cellular immune response estimated through lymphocyte proliferation assay indicated higher levels in CFE than WC dosage. Furthermore, sera obtained after both oral administrations successfully neutralized DENV-1, whereas CFE formulation only neutralized DENV-2 serotype, two representative serotypes which cause severe dengue infection. Sera from mice that were fed CFE preparations demonstrated markedly higher neutralizing titers compared to those from WC-fed mice. However, WC feeding elicited strong immune responses, which were similar to the levels induced by CFE feeding after intraperitoneal booster with purified scEDIII antigen.

**Conclusions:**

CFE preparations of LTB-scEDIII produced strong immunogenicity with low processing requirements, signifying that this fusion protein shows promise as a potent oral vaccine candidate against dengue viral infection.

**Electronic supplementary material:**

The online version of this article (10.1186/s12934-018-0876-0) contains supplementary material, which is available to authorized users.

## Background

Dengue, a dreadful and rapidly spreading arthropod-borne viral disease, affects an estimated 50–400 million people annually [1, 2]. Currently, 2.5 billion people that constitute ~ 40% of the world’s population are at risk of dengue transmission, as it is endemic in at least 100 countries in Asia, the Pacific regions, the Americas, Africa, and the Caribbean. Although the first licensed dengue vaccine CYD-TDV [3] has been recently approved, more potent and effective vaccine developments are still required.

Dengue viruses (DENVs), are positive-stranded RNA viruses belonging to the family *Flaviviridae*. There are four closely related, but antigenically distinct, serotypes (DENV-1, -2, -3, and -4) that can be transmitted to humans through mosquitoes of the genus *Aedes*. Major implications of dengue such as, disease consequences, vaccine induced protection and epidemic immensity depends upon both antigenic and genetic differences among the DENV types [4]. Antibody-dependent enhancement of infection has been proposed as the primary mechanism of dengue immunopathogenesis [5]. Infection by one serotype confers lasting protection against re-infection by the same serotype, however, only transient protection or even detrimental results can occur following the secondary infection [6] by one of the three heterologous serotypes. These findings prompted us to consider a synthetic consensus target sequence from four serotypes to be used as a vaccine candidate.

Dengue infections are categorized as causing asymptomatic fever, dengue fever, and dengue hemorrhagic fever (DHF). DHF can be further classified into four severity grades, with grades III and IV causing dengue shock syndrome with potentially fatal outcomes in the absence of medical care [7, 8]. Despite these differences, each serotype causes nearly identical syndromes in humans and circulates in the same ecological niche. Thus, the ideal dengue vaccine should induce a life-long balanced and lasting immunity against all four DENV serotypes, be free of important reactogenicities, and be affordable.

The DENV envelope (E) protein interacts with several receptors for DENV entry and attachment [9, 10], and is the major protein eliciting a serotype-specific antibody response in the infected host. domain III of the E protein (EDIII) has been implicated in host receptor recognition. EDIII consists of multiple potent and type-specific neutralizing epitopes [11, 12] and acts as an effective antigen to elicit neutralizing antibodies in experimental animal models. This EDIII protein, a polypeptide sequence of ~ 100 amino acids, is an attractive target for the development of a recombinant vaccine [13]. A recent study that developed a consensus EDIII (cEDIII) immunogen by aligning sequences from different isolates of the four serotypes of dengue virus indicated that cEDIII elicits cross-neutralizing antibodies to block DENV1, DENV2, DENV3, and DENV4 infections simultaneously [14]. An engineered heterologous lipoprotein generated by fusing the consensus EDIII protein with lipid signal peptides (LcED III) showed promise in eliciting cellular and humoral immune responses as well as neutralizing antibodies against all four serotypes [15]. In addition, yeast-expressed scEDIII induced balanced immune responses against all four serotypes upon subcutaneous immunization in mice using purified protein emulsified in complete Freund’s adjuvant (CFA) into mice [16].

The concept of oral vaccines has attracted a great deal of attention as they enable a higher capacity for mass immunizations during pandemics at relatively low cost. In addition, they help avoid the risks commonly associated with conventional vaccines, and they confer enhanced immune responses mucosally [17] and systemically. In general, oral vaccines cause less stress and associated immune-suppression for the recipient [17]. Therefore, oral delivery is considered to be an ideal and easy route to introduce foreign antigens. The local antigen presentation in the mucosal tissue is essential for effective tolerance and initiation of active immune responses. Recently, several molecules that could be used to target vaccine antigens to mucosal and systemic compartments have been identified. M cell-specific targeting of the tetravalent dengue antigen (Tet-EDIII) via the Co1 ligand achieved successful targeting to the antigen presenting cells (APCs) of the mucosal immune system [18]. In addition, the B subunit of cholera toxin (CTB) and *Escherichia coli* heat-labile enterotoxin (LTB) are highly efficient carrier molecules for chemically- or genetically-conjugated antigens for eliciting mucosal and systemic antibody responses [19] and mucosal tolerance for prophylactic vaccines against autoimmune diseases [20, 21]. LTB was used in this study not only for its role as an effective adjuvant and carrier of proteins and epitopes, but also for targeting and eliciting the immune response due to the fact that LTB binds with high affinity to its cell surface receptor ganglioside GM1. Ganglioside GM1 binding results in enhanced targeting and access to major histocompatibility complex (MHC) compartments [22, 23], increased activation of APCs and T cells [24], and enhanced stability of the conjugated antigens.

*Saccharomyces cerevisiae* is generally recognized as safe (GRAS). Therefore, it is frequently employed in oral vaccine systems due to the advantage of it being a simple eukaryotic system with high expression capability, ease of scale-up, genetic manipulation, and culturing with the inherent advantage of eukaryotic post-translational modifications and secretion. Moreover, the cells are suitable to be taken up by APCs [25, 26]. The whole recombinant yeast-based vaccine approach integrating efficient antigen delivery with dendritic cell activation without the need for accessory adjuvant components suggests its potential efficiency as an oral vaccination candidate [27]. Furthermore, it has great potential as a system for provoking antigen-specific antibody responses [28]. The use of recombinant *S. cerevisiae* as an oral vaccine and drug delivery system is enhanced by its ability to be absorbed by M cells in the Peyer’s patches (PP) of the gut [29]. Oral administration of freeze-dried *S. cerevisiae* cells expressing the porcine circovirus type 2 (PCV2b) Cap protein on their surface induces protection against subsequent PCV2b challenge; moreover, its properties allow for easier vaccine storage and transport, thus enhancing its attractiveness as a vaccine [30]. Moreover, large amounts of recombinant yeast-producing protein antigens can be easily obtained at low cost. Therefore, strategies using whole recombinant yeast to deliver protein antigens may have the potential for successful antigen delivery and may lead to the induction of cell-mediated immunity directed against a variety of infectious agents.

Taking into consideration the immunogenic efficacy of scEDIII, the efficient mucosal targeting and elicitation of immune responses by LTB, and the fact that *S. cerevisiae* provides an effective vaccine delivery system, we performed a comparative analysis to evaluate the efficacies of dengue virus oral vaccines using WC and CFE preparations of *S. cerevisiae*-expressed *E. coli* LTB-conjugated dengue scEDIII as an oral vaccine candidate in a BALB/c mouse model.

## Methods

### Animal housing and ethics statement

Five-week-old female BALB/c mice procured from the Charles River Laboratories through Orient Bio, Inc. (Sungnam, Korea) and maintained under general specific pathogen-free conditions were used for immunization purposes. Six mice were housed in each filtertop microisolator cage in a temperature- and humidity-controlled room. Mice were acclimated for approximately 1 week prior to the initiation of the oral feeding experiment. Experimental procedures involving laboratory animals strictly adhered to the guidelines set out by the Institutional Animal Care and Use Committee of the Chonbuk National University (Approval Number: CBU 2015-0004).

### Reagents

An anti-dengue virus primary monoclonal antibody (mAb) was procured from LifeSpan BioSciences, Inc., Seattle, WA, USA, and anti-LTB was from Immunology Consultants Laboratory Inc., Portland, OR, USA.

### Construction of an LTB-scEDIII expression vector for expression in *S. cerevisiae*

**Fig. 1:**
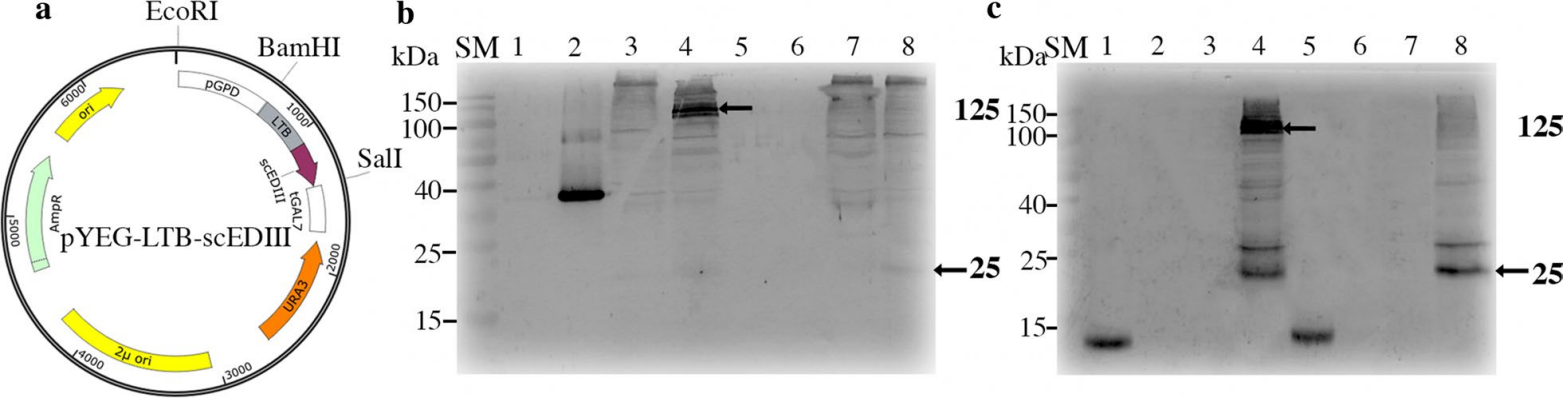
Expression vector construct and expression analysis. **a** Schematic illustration of the expression plasmid pYEG-LTB-scEDIII. Western blot analysis of recombinant *Escherichia coli* heat-labile toxin protein B-subunit (LTB) fused to the consensus dengue envelope domain III (scEDIII) in cell-free extracts (CFEs) using **b** anti-LTB IgG and **c** anti-dengue IgG antibodies under non-denaturing (lanes 1–4) and denaturing conditions (lanes 5–8). Lanes 1 and 5 contain 1 µg of total soluble protein (TSP) extracted from *E. coli*-expressed scEDIII [14] and lanes 2 and 6 contain 1 µg of TSP from *S. cerevisiae*-expressed LTB [32] as positive controls. Lanes 3 and 7 contain 40 µg of TSP extracted from mock-transformed yeast as negative controls. Lanes 4 and 8 contain 40 µg of TSP extracted from recombinant LTB-scEDIII. SM represents protein size marker. Arrows indicate the recombinant LTB-scEDIII protein. The numbers in bold letters on the right indicate the estimated sizes in kDa

LTB and *S. cerevisiae* codon-optimized scEDIII genes [16] were amplified from pYEGLTB [31] and pUC19 harboring scEDIII, respectively. The two resulting polymerase chain reaction (PCR) amplicons were fused by overlap extension PCR using the primers cEIII-LTB OF (5′-GTATGGAAAACGGACCAGGTCCTAAAGGAATGTC-3′) and cEIII-LTB OR (5′-GACATTCCTTTAGGACCTGGTCCGTTTTCCATAC-3′) and DNA polymerase from Takara Bio Inc. In addition, *Bam*HI and *Sal*I restriction sites were included at the 5′ and 3′ ends, respectively, to facilitate subsequent cloning using the primers Con-F (5′-CGGGATCCCGATGAAAGGAATGTCTTACGCA-3′) and Con-R (5′-GACGTCGACGCCTATGAGGAACCCTTTTTAAACCA-3′). The fusion construct was cloned into the yeast episomal shuttle vector, pYEGPD-TER [31] to construct pYEG-LTB-scEDIII with glyceraldehyde-3-phosphate dehydrogenase (GPD) as the promoter and galactose-1-phosphate uridyl transferase (GAL7) as the terminator (Fig. 1a). The resulting clones were confirmed through restriction enzyme digestion and DNA sequencing. The expression host *S. cerevisiae* strain 2805 (*MATα pep4::HIS3 prb1*-*δ Can1 GAL2 his3 ura3*-*52*) [32] was transformed by pYEG-LTB-scEDIII using the lithium acetate method [33]. Empty vector pYEGPD-TER-transformed yeast served as the mock immunization-negative control.

### Northern blot analysis for selecting expression-efficient clones

Total yeast RNAs extracted from the selected clones as described previously [16] were separated on a 1.2% formaldehyde-agarose gel, transferred to an Amersham Hybond™ membrane, and hybridized with an α-[^32^P]-labeled probe generated using a random labeling kit (Amersham Pharmacia Biotech, Piscataway, NJ, USA). The northern blot was developed to visualize the expression pattern of LTB-scEDIII. Expression of the *S. cerevisiae* GPD housekeeping gene served as a reference.

### Culture conditions and target protein expression analysis

The *S. cerevisiae* transformants containing pYEG-LTB-scEDIII were maintained in uracil-deficient (ura^−^) selective medium, (0.67% yeast nitrogen base without amino acids, 0.003% each of adenine and tryptophan, 0.5% casamino acids, and 2% dextrose). Initial seeding was prepared by inoculating 5 mL of ura^−^ medium with a recombinant *S. cerevisiae* colony in a 15-mL Falcon tube, followed by culturing for 48 h at 30 °C with vigorous shaking (200 rpm). The secondary seeding was prepared by inoculating 5 mL of YEPD (1% yeast extract, 2% peptone and 2% dextrose) medium with 250 μL of the primary seeding, followed by culturing for 16 h at 30 °C with continuous agitation (200 rpm). Subsequently, 40 mL of YEPD medium was inoculated with this secondary seeding in a 300-mL Erlenmeyer flask, followed by culturing for 3 day under the same conditions. The cultures were harvested and washed twice with PBS. The fresh weights were recorded, and the samples were stored at − 80 °C for future use. The *E. coli* strains were maintained in Luria–Bertani (LB) broth with the appropriate antibiotics.

For target protein expression analysis, CFEs were prepared as described previously [16]. Protein concentration was determined by Bradford assay using the Bio-Rad Protein Assay Kit (Bio-Rad, Hercules, CA, USA). Sample aliquots of the CFEs were separated by SDS-polyacrylamide gel electrophoresis (SDS-PAGE), and transferred onto Hybond-C extra nitrocellulose filter membranes (Hybond, Amersham Pharmacia Biotech). Primary anti-dengue virus mAb (LifeSpan BioSciences Inc.) and anti-LTB (Immunology Consultants Laboratory Inc.) that recognized scEDIII and LTB, respectively, were used to detect target proteins. After incubation with goat anti-mouse IgG alkaline phosphatase conjugate (Sigma-Aldrich, St. Louis, MO, USA), the blots were developed by BCIP/NBT in TMN buffer (100 mM Tris pH 9.5, 5 mM MgCl_2_, and 100 mM NaCl). Purified protein extract from the BL21 *E. coli* strain expressing scEDIII [16] was used as a positive control.

### Estimation of target protein concentration in CFE

Based on our previous study [34], the amount of recombinant yeast fed to each mouse per dose was determined to be 1.6 g fresh weight of recombinant yeast. To ensure that the equivalent quantities of target protein were fed to all mice, we estimated the amount of target protein in the CFEs from the equivalent weight of cells used as recombinant whole cells. Briefly, CFEs were prepared from 1.6 g wet weight of recombinant yeast cells as described previously [35]. Total soluble protein (TSP) concentration was determined by Bradford assay using the Bio-Rad protein assay kit (Bio-Rad). TSP (40 µg) was coated onto NUNC Maxisorp 96-well enzyme linked immunosorbent assay (ELISA) plates and probed using anti-dengue virus mAb (LifeSpan BioSciences Inc.) to estimate the amount of scEDIII. Total recombinant scEDIII in CFE was quantified based on a standard curve of known amounts of purified *E. coli*-expressed scEDIII [35].

### Preparation of oral doses and quantification of *S. cerevisiae*-expressed LTB-scEDIII

Using the optimal dosage of target protein is necessary to successfully elicit comparable immune responses. According to our previous study, 1.6 g fresh weight of yeast cells was near the maximum dose for oral feeding in mice [34]. We prepared the samples to feed equivalent amounts of recombinant protein irrespective of WC and CFE. Briefly, ~ 20 g fresh weight of recombinant yeast cells was harvested from 640 mL of culture and subsequently divided into two parts: one part was used as WC for oral feeding and the other part was used to prepare CFEs. CFEs from 1.6 g of recombinant yeast cells resulted in a total of 15.5 mg of TSP. The total amount of LTB-scEDIII was estimated using quantitative ELISA. Based on a standard curve of known amounts of purified *E. coli*-expressed scEDIII, we found that 1.3% of TSP corresponded to scEDIII for a total amount of ~ 200 µg of recombinant scEDIII in a single dose.

### Mice immunization and collection of blood and fecal samples

**Fig. 2:**
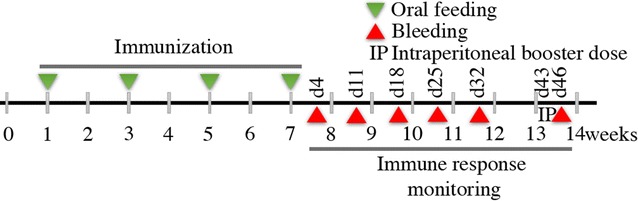
Schematic representation of the oral immunization protocol. Each mouse in a group of six was orally gavaged with either 1.6 g fresh weight of cells resuspended in a final volume of 2.4 mL of PBS or an equivalent amount of TSP in CFE preparations of recombinant yeast cells. For each dose, the WC or CFE suspensions were split equally and administered four times per day. Oral immunization was administered every alternate week up to 4 weeks. Subsequent immune response monitoring was performed through retro-orbital bleeding and fecal matter collection at 4 days after the final feeding and continued every week up to 32 days, followed by intraperitoneal booster injection with purified antigen on day 43 and subsequent bleeding on day 46. ‘d#’ represents days post final vaccination

Prior to oral administration, groups of mice were fasted overnight (water was provided ad libitum). Oral feeding with WC and CFE preparations was performed using a 1-mL syringe fitted with an oral feeding needle. The four groups consisting of six BALB/c mice were fed separately with, Mock WC, LTB-scEDIII WC, Mock CFE, or LTB-scEDIII CFE. Each BALB/c mouse in the WC group was administered 1.6 g fresh weight of cells, resuspended in a final volume of 2.4 mL of PBS, split equally, and orally gavaged four times per dose on every alternate week for 4 weeks as depicted in Fig. 2 with minimum distress. Simultaneously, each mouse in the CFE group was administered the amount of TSP estimated in CFE extracted from 1.6 fresh weight of recombinant yeast cells in a final volume of 2.4 mL of PBS, split equally and orally gavaged four times per dose. To investigate the memory response, an intraperitoneal injection of 20 µg of alum-adsorbed purified *E. coli*-expressed scEDIII was administered to each immunized mouse 3 weeks after the last oral immunization. Blood and fecal pellets were collected periodically from each mouse at day (d)4, d11, d18, d25, and d32 after the last oral immunization (Fig. 2). Blood was collected through retro-orbital bleeding, maintained at room temperature for 1 h to clot, and kept overnight at 4 °C to facilitate clot retraction before serum was recovered for storage at – 20 °C. Extracts from the collected fecal matter were prepared in PBS for ELISA analysis. To observe memory response, weeks after final immunization, intraperitoneal injection of a booster dose of *E. coli* expressed purified scEDIII antigen was given. Then, retro-orbital bleeding was performed after 3 days of injection.

### ELISA detection of anti-dengue antibodies

Changes in humoral immune responses upon oral administration of recombinant WC and CFE were analyzed by estimating the antibodies induced through indirect ELISAs using NUNC Maxisorp 96-well ELISA plates coated with 100 ng/well of *E. coli*-expressed recombinant scEDIII protein. The plates were washed three times with PBS  +  0.05% Tween 20 (PBST) and blocked with 1% BSA in PBS for 2 h at 37 °C. Following the washes, twofold serial dilutions were performed after adding 100 μL/well of sera from immunized mice (the starting dilution points were 1:25 for serum IgG and 1:2 for fecal sIgA), and the plates were incubated overnight at 4 °C. Alkaline phosphatase (AP)-conjugated secondary antibodies (anti-mouse IgG or IgA; Sigma-Aldrich) diluted 1:5000 in PBS containing 0.5% BSA were added and incubated for 2 h at 37 °C followed by a washing step. To detect the response, 100 μL/well of phosphatase substrate (S0942, Sigma-Aldrich) was added and incubated for 15 min at room temperature. The reaction was stopped with 2 M H_2_SO_4_ and the optical density was measured at 405 nm using a microplate reader (Multiskan™ GO Microplate Spectrophotometer, Thermo Scientific Inc., Waltham, MA, USA).

The functional pentameric conformation of yeast expressed LTB-scEDIII was validated for its ability to bind to the GM1-ganglioside receptor using the GM1 ELISA. NUNC Maxisorp 96-well ELISA plates were coated with 3 µg/mL of monosialoganglioside GM1 (G-7641, Sigma-Aldrich) and incubated at 4 °C overnight. The plates were washed three times with PBS  +  0.05% Tween 20 (PBST) and blocked with 1% BSA in PBS for 2 h at 37 °C. Following the washes, the plates were further coated with twofold serial dilutions of *S. cerevisiae*-expressed LTB [38] and LTB-scEDIII CFE and Mock CFE, and then incubated 2 h at 37 °C. Following the washes, GM1 binding was proved using primary antibody (rabbit anti-LTB) and alkaline phosphatase (AP)-conjugated secondary antibodies (anti-rabbit IgG; Sigma-Aldrich) diluted 1:5000 in PBS containing 0.5% BSA followed by the measurement of optical density at 405 nm using a microplate reader following similar steps as mentioned above.

### Lymphocyte proliferation assay

A thymidine incorporation assay as described by Park et al. [36] was used to estimate antigen specific lymphocyte stimulation in isolated lymphocytes of immunized mice. Briefly, isolated lymphocytes (5 × 10^5^) were treated with 2 µg antigen and distributed into each well of a 96-well plate. When the cells in the PBS-treated negative control attained death phase, 0.5 mCi of [^3^H]-TdR (Amersham Life Science, Buckinghamshire, UK) was added to each well to pulse and test for proliferating cells. Stimulation indices (SI) were calculated by dividing the tritium incorporation (cpm), counted using a liquid scintillation counter (Perkin-Elmer, Waltham, MA, USA), in cells treated with antigen by the incorporation in control cells treated with PBS.

### Enzyme-linked immunosorbent spot (ELISPOT) assay

ELISPOT assay were used as described previously [37, 38] to estimate the number of scEDIII-specific IgG or IgA secreting cells in the lymphocytes isolated from spleen, small intestinal lamina propria (LP), and PP of immunized mice. The PPs were carefully excised from the small intestines of mice, and further dissociated into single T cells by stirring with collagenase D (0.5 mg/mL) and DNase I (100 μg/mL) for 60 min at 37 °C. LP cells were prepared by cutting small intestine tissue without PPs into small pieces and then digested with collagenase D (0.5 mg/mL) and DNase I (100 μg/mL) for 60 min at 37 °C. MLN lymphocytes were similarly prepared by the digestion procedure described above.

### DENV neutralization assays

A flow cytometry-based neutralization assay was conducted with U937 + DC-SIGN cells using a protocol similar to that described by Krauss et al. [39]. The DENVs used in this study were kindly provided by Dr. Truong (National Institute of Hygiene and Epidemiology of Vietnam, Hanoi, Vietnam) and propagated in a C6/36 cell line (American Type Culture Collection, Manassas, VA, USA). Briefly, the immune sera collected at d32 after last oral immunization, were serially diluted using five twofold dilutions and incubated with a total of 5 × 10^6^ FACS infectious units (FIUs) of DENV for 1 h at 37 °C under 5% CO_2_. The mixture was then added to the 96-well plate containing 5 × 10^4^ U937 + DC-SIGN cells and incubated at 37 °C under 5% CO_2_. At 2 h post-infection, the cells were washed twice with fresh infection medium and incubated at 37 °C under 5% CO_2_. At 24 h post-infection, the cells were washed, fixed, permeabilized, and stained for DENV E protein using mAB 2H_2_ conjugated to Alexa Fluor 488 (Millipore; Billerica, MA, USA); the percentages of infected cells were measured using flow cytometry. Sigmoidal neutralization curves were generated using GraphPad Prism Mac version 6.0e software. The fluorescence activated cell-based neutralization titer (FNT_50_) of the immune serum was defined as the serum dilution resulting in a 50% reduction in the number of DENV-infected cells.

### Statistical analysis

The statistical significance of the difference between the experimental parameters was determined by two-tailed Student’s *t* test and ANOVA, wherever indicated, using GraphPad Prism Mac version 6.0e. *p*  < 0.05 were considered to indicate significance.

## Results

### Expression of LTB-scEDIII in *S. cerevisiae*

The recombinant plasmid pYEG-LTB-scEDIII harboring the target gene scEDIII under the control of GPD promoter and GAL7 terminator was successfully constructed and transformed into *S. cerevisiae* 2805 strain as described in materials and methods. Eleven presumed recombinant plasmid (pYEG-LTB-scEDIII)-harboring transformants selected from ura^−^ selective medium were further scrutinized with colony PCR and *E. coli* back transformation to confirm the presence of the recombinant plasmid pYEG-LTB-scEDIII. Northern blot analysis of the selected transformants revealed the accumulation of the scEDIII transcript in all transformants (Additional file 1: Figure S1). Among these transformants, one (#5 strain showing the highest transcript level in Additional file 1: Figure S1) was selected for subsequent experiments based on the high expression level of the LTB-scEDIII transcript relative to the internal control (GPD), as evidenced by the densitometric comparison of band intensities (Additional file 1: Table S1).

The selected *S. cerevisiae* transformant strain harboring the pYEG-LTB-scEDIII plasmid was cultured, and production of the target protein was examined using western blot analysis. The presence of LTB-scEDIII fusion protein was confirmed by the cross-reactivity of the protein preparation with anti-LTB anti-dengue antibodies. By probing through both anti LTB and anti scEDIII, it showed the presence of the LTB-scEDIII protein band at ~ 125 kDa under non-denaturing conditions and at approximately 25 kDa under heat-denaturing conditions (Fig. 1b, c).

### Functional pentameric conformation of *S. cerevisiae* expressed LTB-scEDIII

The pentameric conformations of LTB is essential for GM1 ganglioside receptor binding which is vital for antigen uptake into mucosal system. Thus, we analyzed the oligomeric conformation of LTB-scEDIII by western blot analysis using anti-LTB and anti-dengue antibodies. Western blot analyses revealed the presence of the LTB-scEDIII protein band at ~ 125 kDa under non-denaturing conditions and at approximately 25 kDa under heat-denaturing conditions (Fig. 1b, c). These results suggested that the LTB-scEDIII fusion protein assembled into a pentameric form under native conditions, which is essential for binding the GM1 ganglioside receptor. This functional pentameric conformation was further validated for its ability to bind to the GM1-ganglioside receptor using the GM1 ELISA (Additional file 1: Figure S2). The results of the GM1 ELISA indicated that the LTB-scEDIII fusion protein exhibited a strong affinity to GM1 gangliosides, whereas the mock transformants showed no affinity to GM1 gangliosides. These results strongly suggest that the LTB-scEDIII fusion protein assembled into its oligomeric structure without hindrance from scEDIII. Furthermore, it maintained its functionality of receptor binding, which is required for target protein entry via ligand-specific binding activity of the pentameric LTB.

### Evaluation of the systemic humoral immune response elicited upon oral immunization with recombinant LTB-scEDIII in WCs and CFEs

**Fig. 3:**
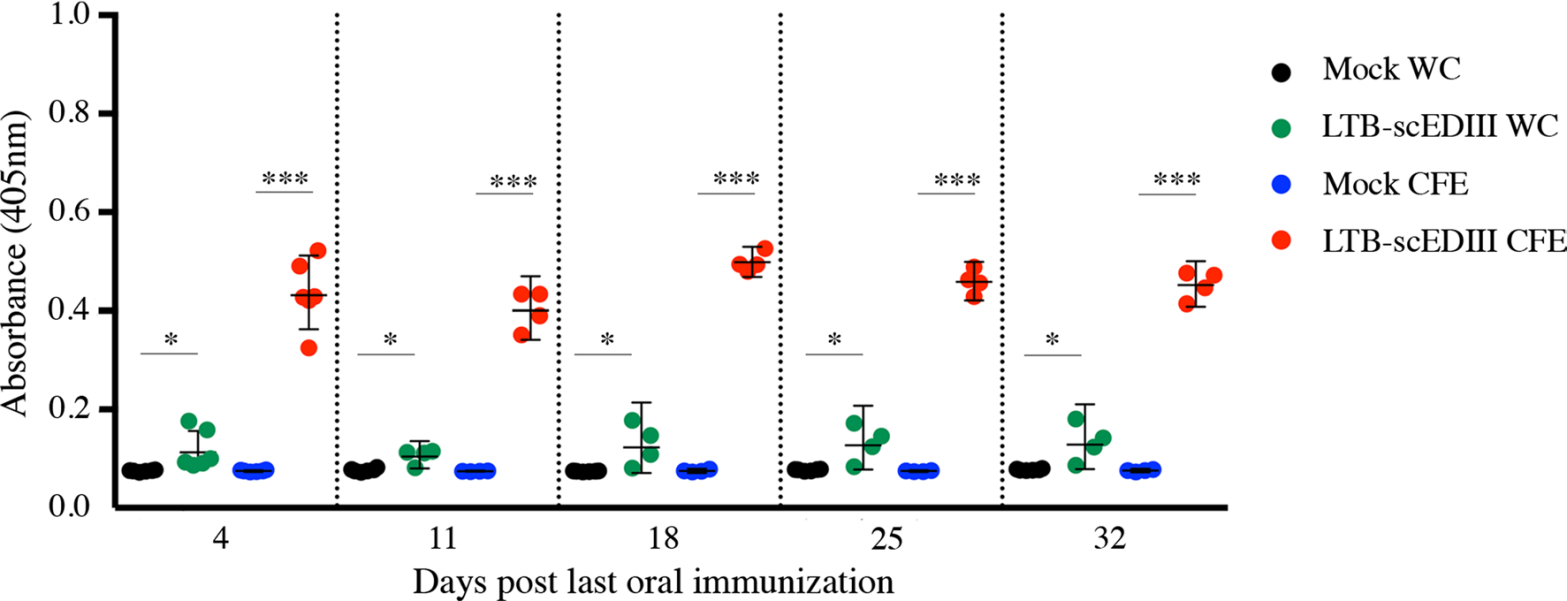
Humoral IgG responses in orally immunized mice. scEDIII-specific serum IgG was induced in mice that received oral administration of LTB-scEDIII WC and LTB-scEDIII CFE, and this induction was observed at 4 days after the last immunization until day 32, as measured at weekly intervals. An unpaired Student’s *t* test was used to calculate *p* values, and *p < 0.05 and ***p < 0.001 indicate significant differences between the groups

LTB is an effective mucosal adjuvant and *S. cerevisiae* cells have multiple adjuvant properties, making them an ideal combination to elicit both cellular and humoral immune responses. Thus, we explored the immunogenicity of yeast-expressed LTB-scEDIII through oral administration of two dosage forms: WCs (LTB-scEDIII WC) and CFEs (LTB-scEDIII CFE). Mice immunized with LTB-scEDIII WC and LTB-scEDIII CFE revealed significantly higher scEDIII-specific IgG antibody titers compared to Mock WC and Mock CFE obtained from mock transformants (Fig. 3). The immune responses showed prime-boost-dependent kinetics, with antibody titer sharply rising following the second boost. However, mice immunized with LTB-scEDIII WC showed lower levels of scEDIII-specific IgG compared with those immunized with LTB-scEDIII CFE. No scEDIII-specific IgG antibodies were detectable in the mice administered with Mock CFE and Mock WC.

### Evaluation of the mucosal immune response elicited upon oral immunization with recombinant LTB-scEDIII in WCs and CFEs

**Fig. 4:**
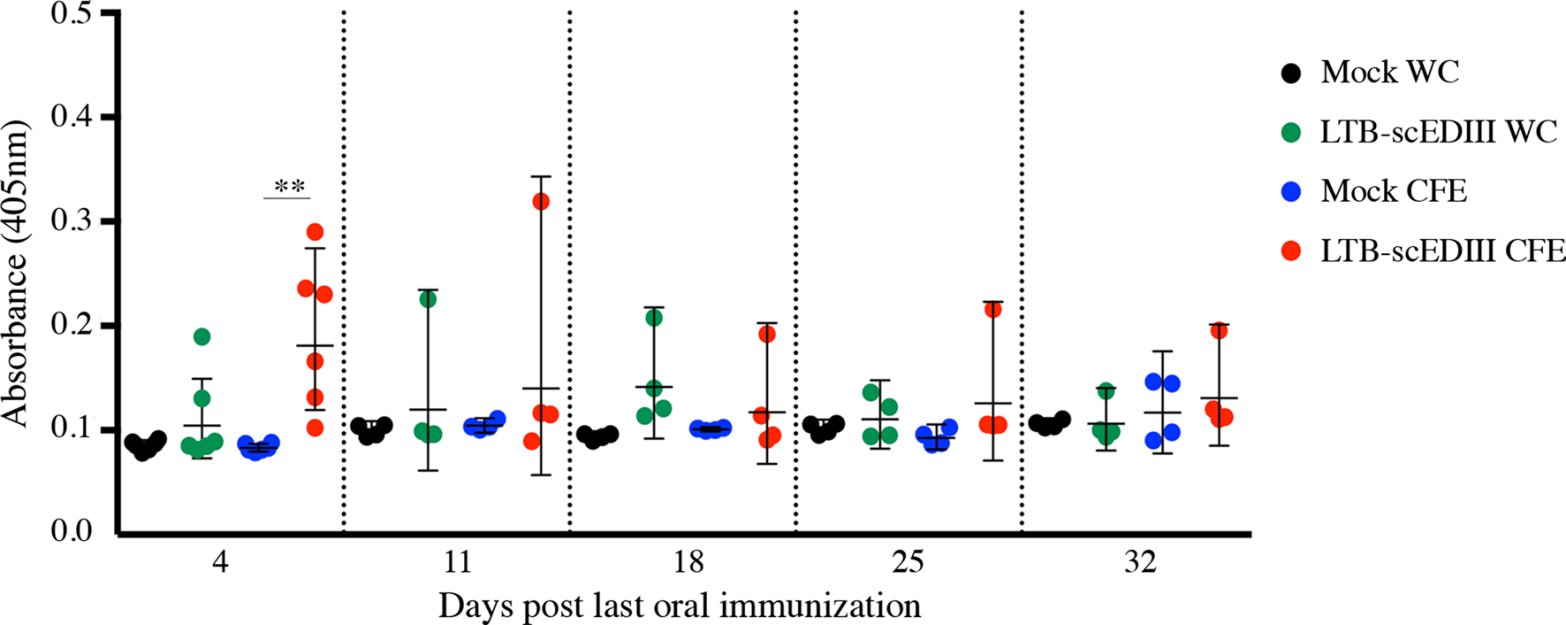
Mucosal sIgA responses upon oral immunization. scEDIII-specific fecal sIgA was induced in mice immunized orally with recombinant LTB-scEDIII WC and LTB-scEDIII CFE at 4 days after the last immunization until day 32, observed at weekly intervals. An unpaired Student’s *t* test was used to calculate *p*-values, and *p < 0.05 and **p < 0.01 indicate significant differences between the groups

The mucosal immune response evaluated by fecal sIgA antibody titers with ELISA showed that mice immunized with LTB-scEDIII CFE or LTB-scEDIII WC elicited scEDIII-specific fecal sIgA; however, mice immunized with LTB-scEDIII CFE demonstrated higher levels than those immunized with LTB-scEDIII WC (Fig. 4). These results suggest that orally administered LTB-scEDIII is targeted to the intestinal lymphoid tissues where it induces mucosal antibody responses.

**Fig. 5:**
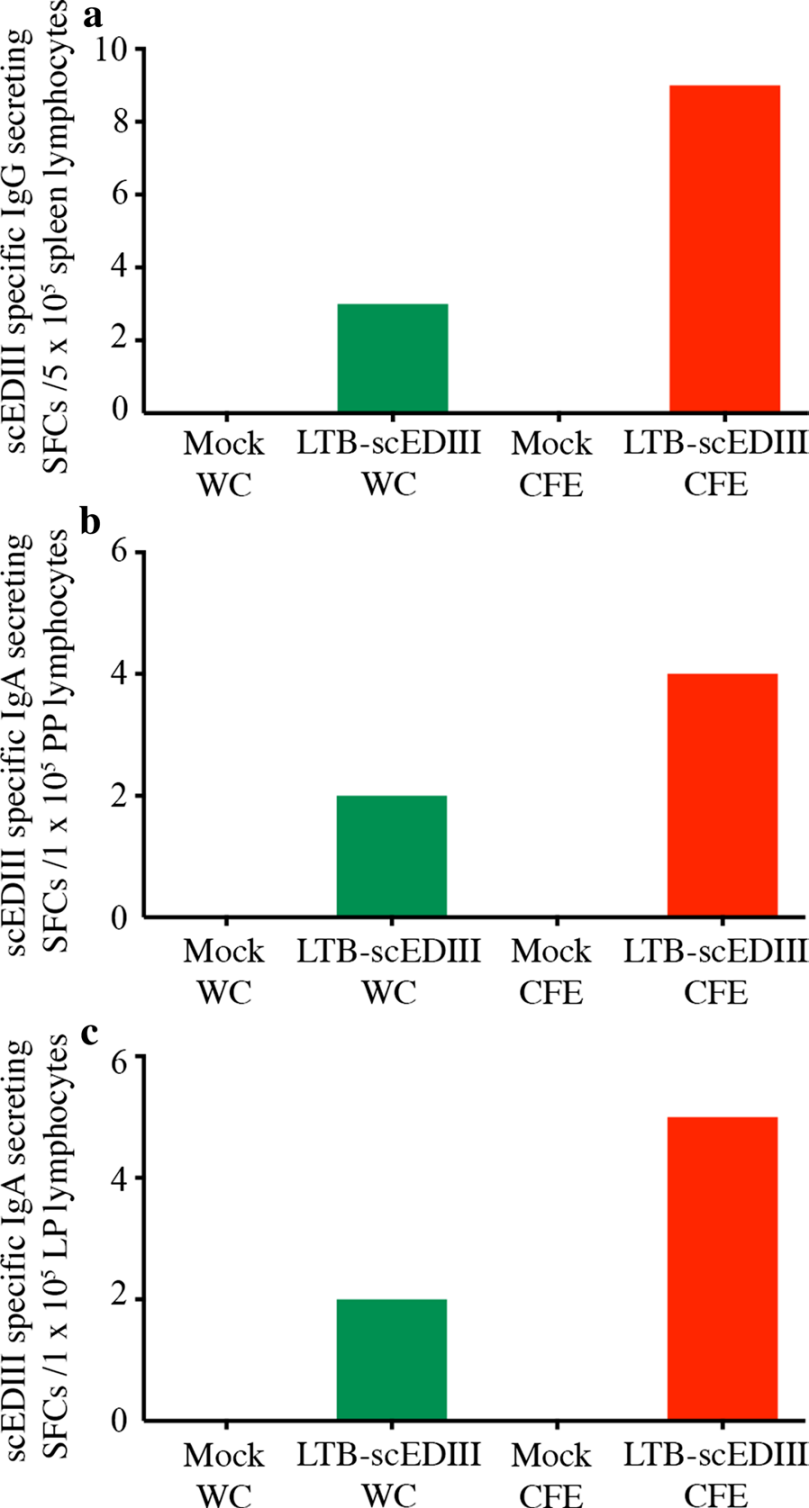
Frequency of antigen-specific immune cells in orally immunized mice. ELISPOT assay showing scEDIII-specific IgG and IgA antibody spot forming cells in the lymphocytes isolated from spleens, LP, and PP of immunized mice. Each group represents two mice analyzed individually in triplicates. This is a representative result of two independent experiments showing similar results

To further confirm the scEDIII-specific mucosal immune responses, we performed ELISPOT assays on lymphocytes isolated from mucosal tissues such as spleen, LP, and PP from immunized mice. Briefly, 7 days after the last booster vaccination, two mice were sacrificed from each group, and their spleens, LP, and PP were retrieved. Lymphocytes were isolated from the tissues and probed for scEDIII-specific IgG- and IgA-secreting cells through ELISPOT assays. The number of scEDIII-specific antibody forming cells (AFCs) in the form of spot-forming cells (SFCs) in the spleen, LP, and PP lymphocytes differed considerably between LTB-scEDIII-administered mice and their Mock-administered counterparts, irrespective of dosage type (Fig. 5). Furthermore, comparatively higher numbers of scEDIII-specific IgG AFCs in splenic lymphocytes and scEDIII-specific IgA AFCs in the PP and LP lymphocytes were observed in the LTB-scEDIII CFE than in the LTB-scEDIII WC mice. The mucosal cells from Mock-fed mice showed no scEDIII-specific IgG AFCs or IgA AFCs in mucosal cells. After booster stimulation with scEDIII purified antigen, lymphocytes were collected and analysed. Comparatively higher numbers of scEDIII-specific IgG AFCs in splenic lymphocytes were observed after administration of antigenic booster dose (Additional file 1: Figure S3). These results revealed that the enhanced mucosal immune responses were due to the oral administration of LTB-scEDIII, suggesting that the enhanced levels of scEDIII-specific serum IgG and fecal IgA were caused by the efficient targeting to the mucosal immune system due to the presence of LTB on the fusion protein.

### Elevation of cellular immune responses with LTB-scEDIII oral immunization

**Fig. 6:**
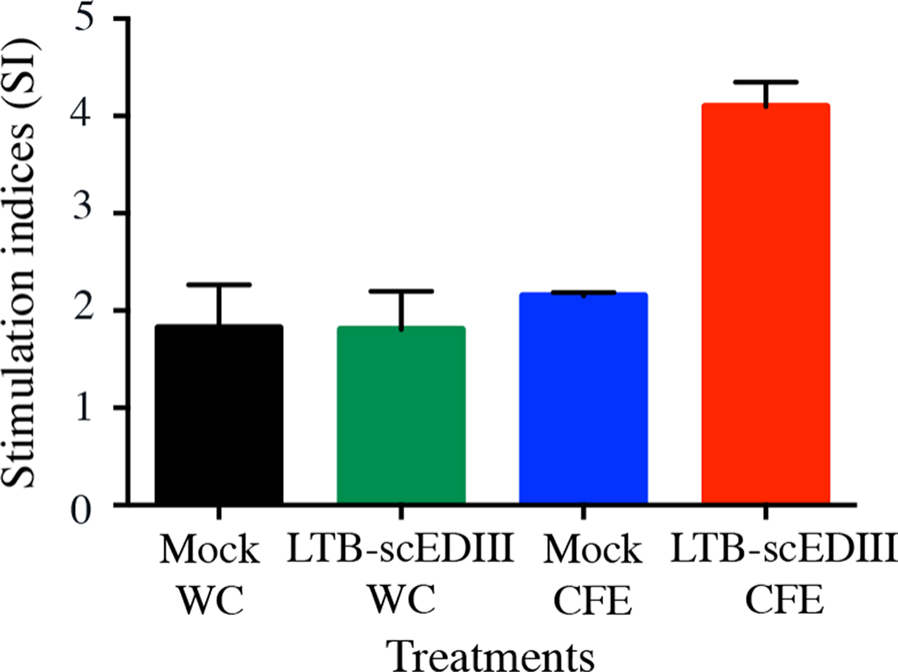
Cellular immune responses upon oral immunization. Cellular immune responses represented by lymphocyte proliferation was determined from [^3^H]-TdR incorporation after in vitro stimulation of isolated lymphocytes with *E. coli* expressed scEDIII, and results are expressed as stimulation indices. All assays were performed in triplicates and the values (from a representative experiment of typically three performed) are shown as the mean ± standard deviation

Although oral immunization with recombinant LTB-scEDIII WC and CFE induced dengue_1_-specific serum IgG and mucosal sIgA production, cell-mediated immune responses also play a crucial role in protecting the host from invading pathogens. Therefore, we evaluated the proliferative response of isolated splenic lymphocytes primed with the scEDIII antigen in vitro to determine the systemic cellular immune response to LTB-scEDIII oral immunization. The SI of the lymphocytes from CFE immunized mice was significantly higher (p < 0.05) than those of the WC and Mock. However, lymphocytes from WC immunized mice revealed similar proliferative activity with that of Mock immunized mice. These results suggested that LTB-scEDIII CFE oral immunization resulted in better cellular immune response than that of WC (Fig. 6).

### LTB-scEDIII induced EDIII-specific neutralizing antibodies

**Fig. 7:**
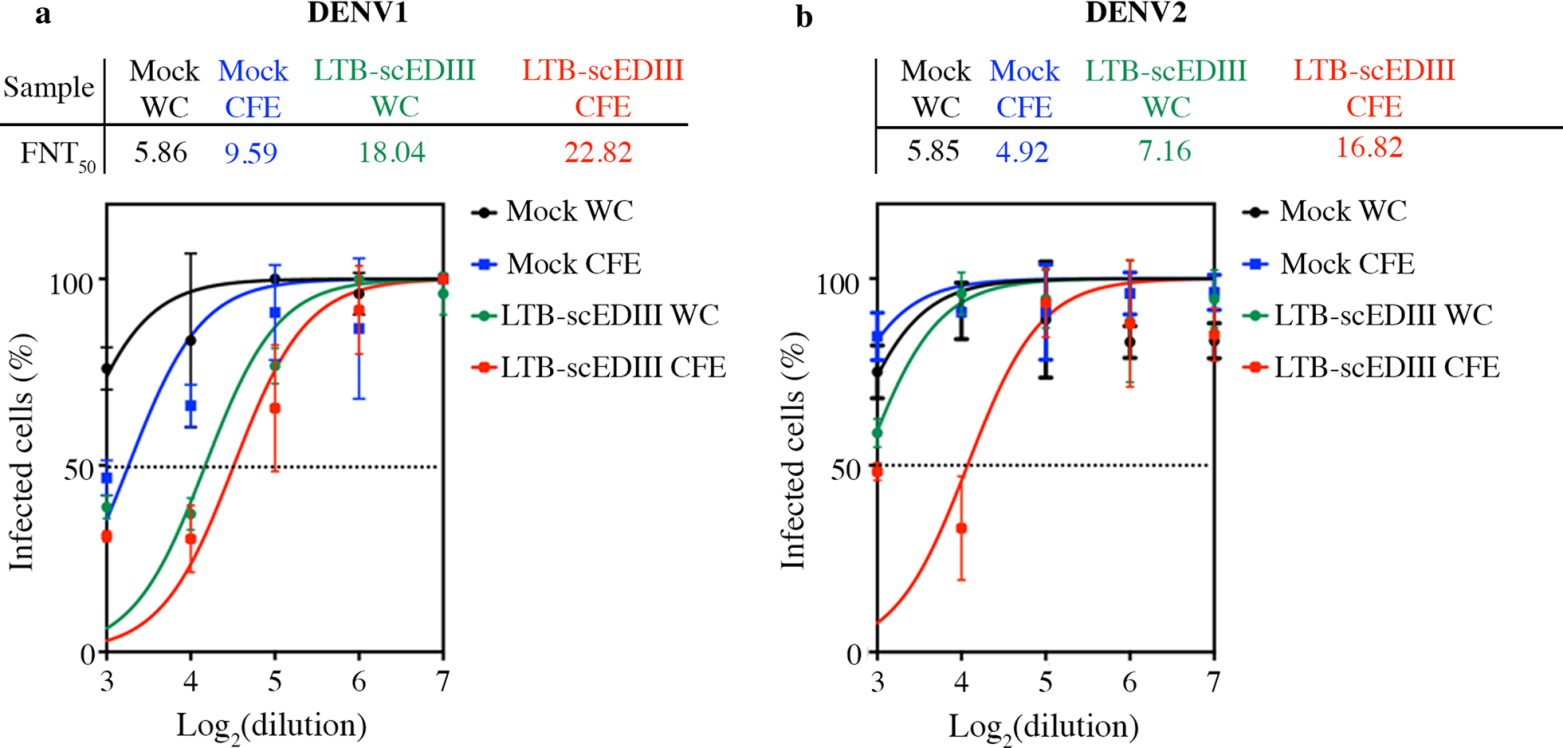
Characterization of neutralization potency of LTB-scEDIII-induced antibodies. Twofold serial dilutions of heat inactivated immune serum from LTB-scEDIII-immunized mice, collected at d32 after last oral immunization were assessed for their potency to neutralize and inhibit the infectivity of **a** DENV-1 and **b** DENV-2 using a FACS-based neutralization assay. The y-axis corresponds to the observed percentage of virus infection. The dotted horizontal line represents 50% infection. The x-axis corresponds to the logarithm of serum dilution. The neutralization assay was performed in triplicates and the values (from a representative experiment of typically three performed) are shown as the mean ± standard deviation

A major goal in dengue vaccine development is to obtain sufficient neutralizing efficiency of the candidate vaccine against all serotypes of dengue virus. Following the preliminary analysis of the antibodies elicited by oral immunization with LTB-scEDIII, we next sought to determine whether these could prevent DENV from infecting susceptible cells. Thus, we employed a FACS-based virus neutralization assay against DENV1 and DENV2, which are the two representative serotypes responsible for severe dengue infection. The neutralization assay results revealed that sera from mice orally immunized with LTB-scEDIII CFE or LTB-scEDIII WC neutralized DENV1, whereas LTB-scEDIII CFE rather than LTB-scEDIII WC could only neutralize DENV2 (Fig. 7). However, the neutralization titers of sera from mice immunized with LTB-scEDIII CFE were comparatively higher than those of sera from mice immunized with LTB-scEDIII WC. The LTB-scEDIII CFE-derived antibodies successfully neutralized DENV1 with FNT_50_ titers > 22 and DENV2 with FNT_50_ titers > 16. LTB-scEDIII WC-derived antibody showed DENV1 FNT_50_ titers > 18; however, significantly lower titers were observed with DENV2 (FNT_50_ > 7), which suggests the inability of WC formulation in neutralizing DENV2.

### Oral immunization elicits memory response upon booster immunization

**Fig. 8:**
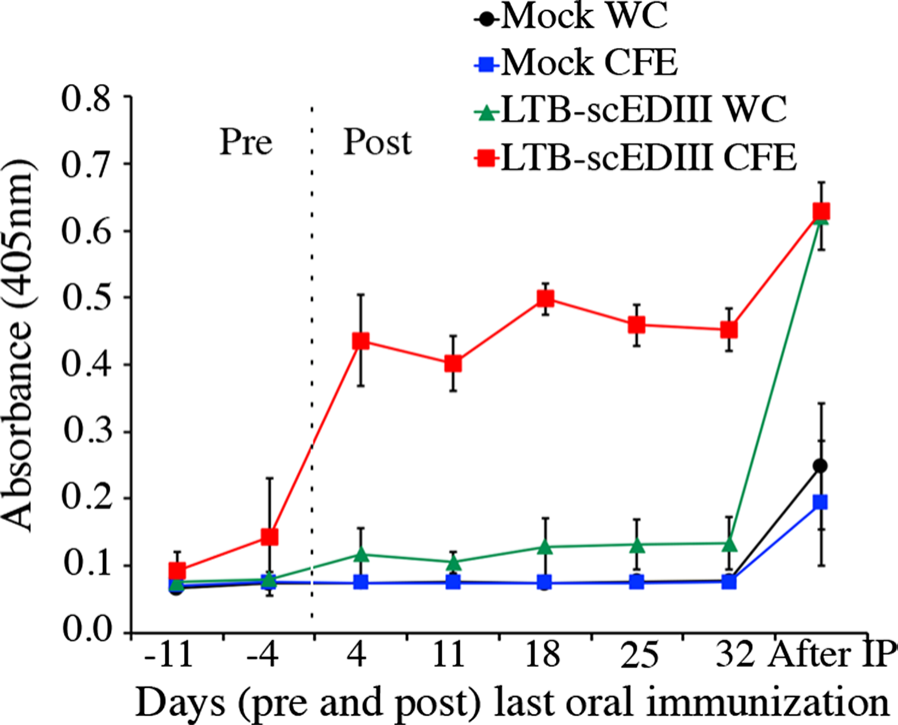
Memory response upon intraperitoneal booster immunization in orally immunized mice. Comparative analysis of serum IgG levels pre- and post-immunization and after intra-peritoneal booster injection using 20 µg of purified *E. coli*-expressed scEDIII in each immunized mouse. A strong memory response was elicited upon booster immunization in both WC- and CFE-immunized mice. All assays were performed in triplicates, with pooled serum samples from each week and the values (from a representative experiment of typically three performed) are shown as the mean ± standard deviation

An important feature of a vaccine is its ability to induce a long-term memory response to antigenic challenge. To determine whether memory immune cells had been established as a result of oral immunization, we evaluated serum IgG levels after an intra-peritoneal booster injection of 20 µg of alum-adsorbed purified *E. coli*-expressed scEDIII was administered to each immunized mouse on day 43 following the last oral immunization. Retro-orbital bleedings were conducted after 3 days to collect serum for IgG analysis. Interestingly, although the immune response due to recombinant WC was considerably lower than that induced by recombinant CFE, the secondary immune response of recombinant WC surged and matched that of recombinant CFE upon intraperitoneal antigenic booster dose (Fig. 8). The IgG titers in both LTB-scEDIII CFE and LTB-scEDIII WC were significantly higher than those of the booster dose immunized Mock CFE and Mock WC, respectively. These results predict that a strong memory response might have been elicited upon oral immunization with LTB-scEDIII regardless of the WC and CFE preparation.

## Discussion

In this study, we evaluated the efficacy of a dengue oral vaccine candidate administered to mice using WC and CFE preparations. We previously confirmed that yeast-expressed scEDIII resulted in a balanced immune response against all four serotypes [16], suggesting its role as a promising tetravalent dengue vaccine candidate. Furthermore, since LTB displays advantageous adjuvant properties as a co-administered or conjugated antigen [40, 41], we investigated the immunogenicity and neutralization capacity of the oral vaccine consisting of yeast cells expressing the LTB-scEDIII fusion protein.

Before utilizing the *S. cerevisiae*-expressed LTB-scEDIII as an oral vaccine candidate, we determined whether the antigenic integrity of scEDIII was maintained, despite being fused to LTB. As expected, the recombinant protein preserved its antigenic identity, as it was detected by western blotting and indirect ELISA probed with anti-dengue mAb. These results were consistent with previous findings, which showed that the dengue scEDIII fusion protein was expressed successfully in plant and yeast systems, preserving its antigenic properties [31, 42, 43]. The functionality of LTB as a potent immunoadjuvant depends on the pentameric formation of LTB, which is required for binding with GM1 ganglioside. Using a GM1 ELISA, we found that the LTB-scEDIII recombinant protein retained its biological ability to form pentameric associations. Previous studies reported that bacterial LTB subunits assembled into functional pentameric proteins in plants and yeast [44–46]. However, steric hindrance from large molecules fused with LTB might prevent pentamer formation [47, 48], suggesting that smaller targets or co-expression would be required for pentamer formation [34]. In our study, as scEDIII is a smaller protein, no such obstacles were observed. The yeast-expressed LTB-scEDIII followed a similar pattern of GM1 binding as that of the positive control yeast expressing LTB, further proving its functional effectiveness.

After confirming the functional characteristics of the recombinant LTB-scEDIII protein, we investigated its immunogenicity. We found that all groups that received the recombinant protein using WC and CFE preparations developed anti-dengue IgG titers at 4 days after the last immunization, showing titers that differed significantly from controls. Similar observations were reported in a study using plant-based dengue oral immunization with CTB-sEDIII [49]. Mucosal vaccines given via the oral route can induce mucosal immunity, such as antigen-specific sIgA production, as well as systemic immunity [50]. In addition to systemic immunity, mucosal immunity was also elevated. Elevated fecal sIgA levels in both recombinant yeast WC- and CFE-fed mice apparently reflected enhanced stimulation by exposure of PP and LP to the same antigen LTB-scEDIII. Although the sIgA titers were low, upon further analysis, the numbers of AFCs in the lymphocytes isolated from spleen, LP, and PP were found to differ considerably between LTB-scEDIII-administered mice and their Mock-administered counterparts, irrespective of dosage preparation. Cellular immune responses are simultaneously essential in imparting protection against DENV infection. Although, the lack of DENV non-structural (NS) proteins, a source of T cell epitopes, in our scEDIII construct questions the ability to elicit T cell immune response that affects long-term immunogenicity and protection, previous studies have demonstrated the successful activation of T cells using DENV EDIII as a target. T cells from SPLs of EDIII-Co1A immunized mice successfully secreted cytokines such as IL-2, IL-4, IL-6 and IL-17 after in vitro EDIII stimulation [51]. The consensus EDIII expressed in both tobacco plants was reported to have induced a significant cellular immune response, in the form of IFNγ production and polyfunctional T cells in both the CD4+ and CD8+ compartments [52]. In this study also, cellular immune response in the form of lymphocyte proliferative activity was elevated in CFE-fed mice. The overall limited efficacy of oral vaccines is both due to problems linked to antigen breakdown in the harsh gastric environment and also to the highly tolerogenic gut environment. The relative elicitation of an immune response confirmed the suppression of oral tolerance, a property that most LTB- and CTB-fused antigens reveal when administered without priming [53, 54].

Next, we evaluated the potential of the yeast derived LTB-scEDIII as an oral vaccine using WC and CFE preparations. Vaccines delivered orally are processed and presented in the mucosal system, thus targeted immunization requires efficient adjuvants. The CTB subunit from *Vibrio cholerae* and the LTB subunit of *E. coli* are well-characterized mucosal carrier proteins [34, 48]. *Saccharomyces cerevisiae* is considered to be GRAS and is used as a food and feed ingredient [55, 56]. It is suitably taken up by APCs [25, 26], and the immunostimulatory effects of the cell wall components have been reported in several yeast strains [57–59]. In the current study, the LTB-scEDIII antigen was expressed in *S. cerevisiae*, it folded efficiently into the biologically active pentameric form that interacted with GM1-ganglioside, and it was recognized by anti-LTB IgG. Oral immunization with WC or CFE preparations elicited potent humoral immune responses systemically and in the mucosa, as confirmed by the significantly high serum IgG and fecal sIgA levels in serum and fecal extracts, respectively, further justifying its use as an oral vaccine candidate.

Establishment of a memory response is an important criterion in designing and implementing an effective vaccine. Our studies revealed that feeding recombinant LTB-scEDIII WC or CFE to mice stimulated a primary immune response. Moreover, several weeks after the decline of specific antibodies to background levels, a single intra-peritoneal injection of purified scEDIII provoked a rapid surge in an immune response, especially in the WC-fed mice. The responses to the antigenic boost were significantly lower in mice that were fed with WC and CFE derived from Mock-transformed cells, showing that the boosting response elicited in mice fed with recombinant LTB-scEDIII WC or CFE was indeed the result of priming and establishment of immune memory to LTB-scEDIII presented in the gut. Although, further analysis through ELISPOT assay could not confirm the elevated immune response against LTB-scEDIII WC formulation, but LTB-scEDIII CFE immunized mice indicated significantly increased immune response (> fourfold rise) upon booster immunization. The level of the immune response to a booster immunization gets significantly elevated indicating vaccines can still rely on a significant local immunity that may interfere with colonization of the gut by the vaccine strain for a long time after basic immunization [60]. These data further indicate that LTB-scEDIII, produced and delivered via CFE dosage preparations, is an effective oral immunogen, representing a potent dengue oral vaccine candidate.

The ideal dengue vaccine should be free of significant reactogenicity, induce a life-long balanced and lasting immunity against all four DENV serotypes, and be affordable. Therefore, it should be capable of neutralizing DENV serotypes in a cost-effective manner. DENV EDIII is involved in receptor binding. It is the target of specific neutralizing antibodies, and is considered to be a promising subunit dengue vaccine candidate [61]. Our study using the FACS-based neutralization assay revealed that the antibodies produced against scEDIII through oral immunization can neutralize DENV1 and DENV2 serotypes, the representative serotypes causing severe dengue infection. Earlier reports indicated that DENV1 and DENV2 were the predominant serotypes in a case study in Singapore [62]. Studies in Thailand and Taiwan found a significant correlation between DENV-2 and greater disease severity [63]. DENV2 induced increased disease severity, which was correlated with high viremia titer and secondary dengue infection [64]. In our study, the neutralization titers were higher in CFE-fed mice compared with WC-fed mice.

Antigen delivery by oral route is thought to be an ideal strategy for vaccination. Oral vaccines are particularly attractive for immunization in developing countries as they enable mass vaccination at relatively low cost, help avoid the risks commonly associated with conventional vaccines, and confer enhanced immune responses at mucosal sites and systemically [65, 66]. Dengue is listed as a neglected tropical disease, and apart from the technical constraints, the costs associated with mass immunization, maintenance of a sustained supply, and potential safety issues are major concerns in its eradication [6, 67]. The current study presents a cost-effective oral vaccination strategy for administering *S. cerevisiae*-expressed LTB-scEDIII in the form of WC or CFE. This strategy does not require intricate processing, it elicits humoral and cell-mediated immune responses, and stimulates neutralizing antibodies against dengue virus serotypes.

Although both dosage forms of the evaluated dengue vaccine strategy elicited humoral and cellular immune response with high neutralizing efficiency and strong memory responses, immunization with WC elicited a lowered immune response and neutralization efficiency in comparison with CFE. This lower response may be caused by improper targeting. The yeast cell surface is a functional interface where natively displayed molecules on the surface play important roles in various biological or physiological processes [68]. Thus, the strategy of displaying target proteins on the yeast surface is currently used as an efficient vaccine delivery system [69, 70]. Therefore, the elicited immune response due to oral administration of LTB-scEDIII WC could be improved through the surface display of scEDIII, thus enhancing mucosal targeting of the target antigen. Future studies should investigate the advantages of displaying antigen on the yeast surface in the development of efficient oral dengue vaccines.

## Conclusions

This study is the first to report the potential of *S. cerevisiae*-expressed LTB-scEDIII as an oral vaccine candidate in the form of WC or CFE. Comparing the immunogenicity of WC and CFE vaccine delivery, oral immunization with CFE provoked higher humoral and cellular immune responses as well as higher neutralizing titers. However, WC-immunized mice showed a stronger memory response upon intraperitoneal booster dose. These findings suggest that the LTB-scEDIII fusion protein delivered in the form of CFE rather than WC shows promise as a potent oral vaccine candidate against dengue virus infection.

## Additional file

**Additional file 1.** Comparative immunogenicity of preparations of yeast-derived dengue oral vaccine candidate.

## Abbreviations

DENV: dengue virus
WC: whole recombinant yeast cells
CFE: cell-free extracts
LTB: heat-labile toxin protein B-subunit
scEDIII: consensus dengue envelope domain III
DHF: dengue hemorrhagic fever
CFA: complete Freund’s adjuvant
APCs: antigen presenting cells
MHC: major histocompatibility complex
GRAS: generally regarded as safe
PP: Peyer’s patches
LP: lamina propria
PCV2b: porcine circovirus type 2
ura^−^: uracil-deficient
SDS-PAGE: SDS–polyacrylamide gel electrophoresis
TSP: total soluble protein
ELISA: enzyme linked immunosorbent assay
SI: stimulation indices
ELISPOT: enzyme-linked immunosorbent spot
