# Supplementary material for: Comparative immunogenicity of preparations of yeast-derived dengue oral vaccine candidate
Source: Microb Cell Fact. 2018 Feb 16;17:24. doi: 10.1186/s12934-018-0876-0 (PMC5815244; doi:10.1186/s12934-018-0876-0)
Supplement: Supplementary file 1 — Additional file 1. Comparative immunogenicity of preparations of yeast-derived dengue oral vaccine candidate. [file 12934_2018_876_MOESM1_ESM.docx]

Additional information

Comparative Immunogenicity of Preparations of Yeast-Derived Dengue Oral Vaccine Candidate

Jyotiranjan Bal^1^, Nguyen Ngoc Luong^2^, Jisang Park^1^, Ki-Duk Song^3^, Yong-Suk Jang^1^, and Dae-Hyuk Kim^1^*

^1^Department of Molecular Biology, Department of Bioactive Material Sciences, Institute for Molecular Biology and Genetics, Chonbuk National University, Jeonju, Jeollabuk-do, 54896, Republic of Korea.

^2^Department of Biology, College of Sciences, Hue University, Vietnam

^3^Department of Animal Biotechnology, The Animal Molecular Genetics and Breeding Center, Chonbuk National University, Jeonju, Jeollabuk-do, 54896, Republic of Korea.

*** Correspondence:** Dae-Hyuk Kim: [dhkim@jbnu.ac.kr](mailto:dhkim@jbnu.ac.kr)

Table of contents

[Figure S1 2](#_Toc496879350)

[Figure S2 3](#_Toc496879351)

[Figure S3 4](#_Toc496879352)

Table S1..........................................................................................................................5

References.......................................................................................................................6

## Figure S1





Figure S1. Northern blot analysis of LTB-scEDIII transcripts from 11 selected transformants. Lanes 1 and 2 contain plasmid DNA of pYEG-GPD and pYEG-LTB-scEDIII, respectively, as hybridization controls. Lane 3 contains RNA from mock-transformed cells. Lanes 4–14 contain 30 µg of total RNA from transformants #1–11, respectively. Glyceraldehyde-3-phosphate dehydrogenase (GPD) transcription is shown as an internal control. Note that lane 2 shows the hybridizing band because the GPD probe was constructed using the complete GPD gene, including a part of the promoter as a template. Ribosomal RNA (rRNA) is shown to verify that an equal amount of RNA was loaded in each lane.

## Figure S2


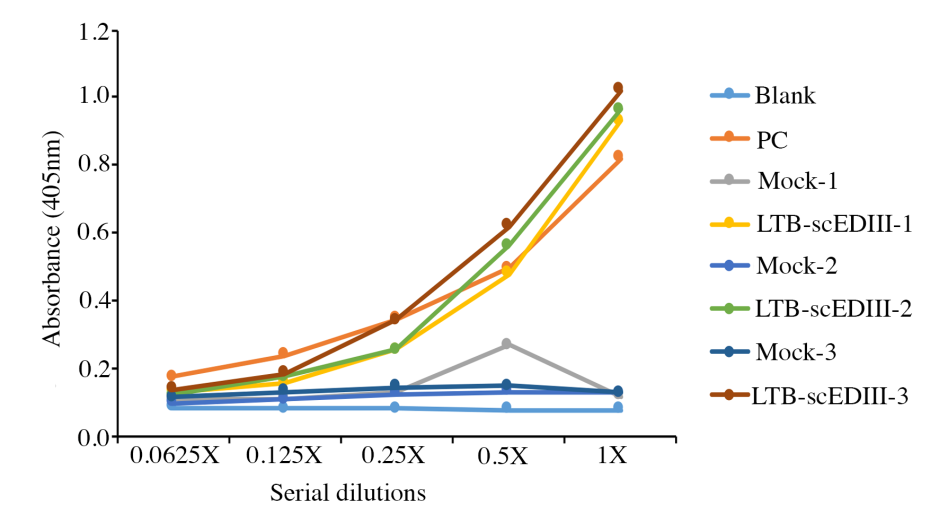


Figure S2. GM1 ELISA confirmation of the functional pentamerization of LTB-scEDIII. Equal concentrations of LTB-scEDIII CFE and Mock CFE with decreasing two-fold dilutions were analyzed for ganglioside GM1 ELISA binding. Positive control (PC) represents purified *S. cerevisiae-*expressed LTB [1]

## Figure S3
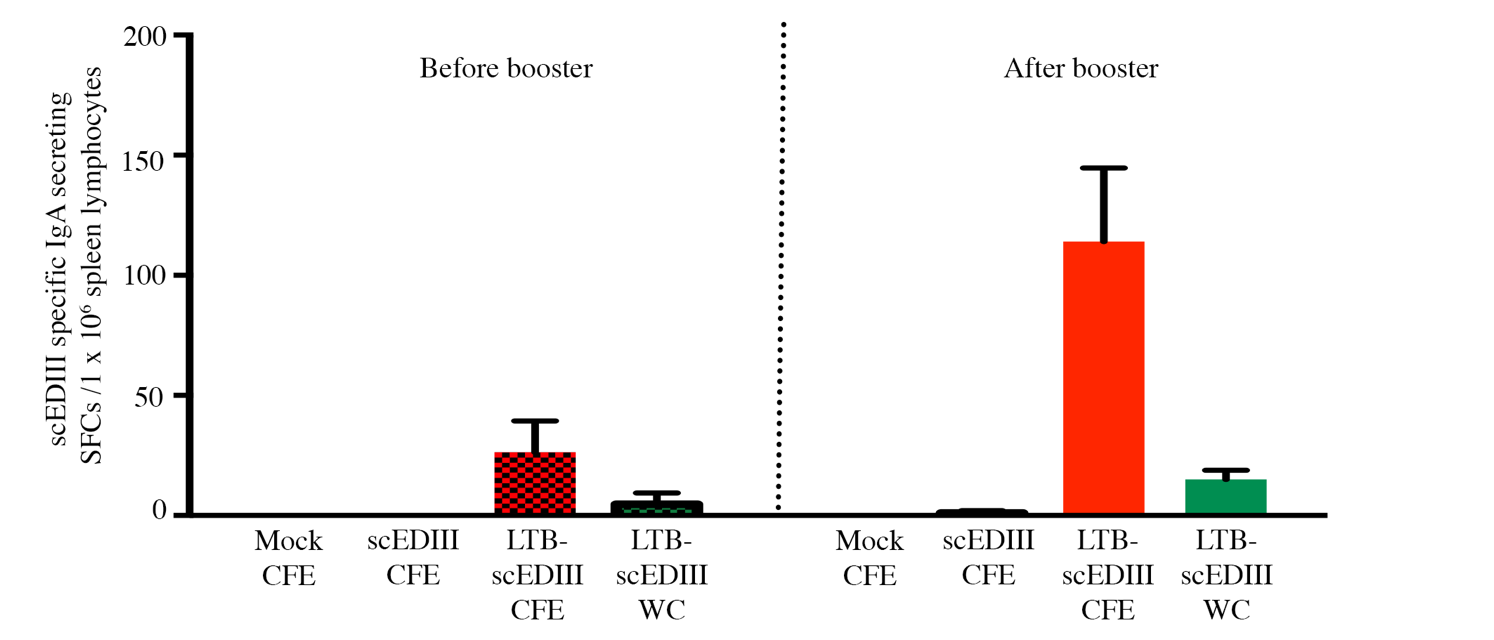


Figure S3. Frequency of antigen-specific immune cells before and after antigen stimulation . ELISPOT assay showing scEDIII-specific IgG antibody spot forming cells in the lymphocytes isolated from spleens of immunized mice, before and after booster dose stimulation Each group represents three mice analyzed individually in triplicates.

Table S1. Densitometric comparision of expression levels of LTB-scEDIII in the transformants

| **Strain #** | **Gene of interest** | **Area** | **Ratio** |
| --- | --- | --- | --- |
| 1 | LTB-scEDIII | 73492.668 | 1.121 |
|  | GPD | 65538.973 |  |
| 2 | LTB-scEDIII | 82545.345 | 1.161 |
|  | GPD | 71119.872 |  |
| 3 | LTB-scEDIII | 88110.910 | 1.130 |
|  | GPD | 77953.751 |  |
| 4 | LTB-scEDIII | 86259.617 | 1.118 |
|  | GPD | 77154.094 |  |
| 5 | LTB-scEDIII | 140462.082 | 1.241 |
|  | GPD | 113183.508 |  |
| 6 | LTB-scEDIII | 119665.366 | 1.043 |
|  | GPD | 114691.085 |  |
| 7 | LTB-scEDIII | 119114.739 | 1.101 |
|  | GPD | 108180.671 |  |
| 8 | LTB-scEDIII | 59298.818 | 0.910 |
|  | GPD | 65188.538 |  |
| 9 | LTB-scEDIII | 57292.061 | 1.024 |
|  | GPD | 55973.295 |  |
| 10 | LTB-scEDIII | 55464.768 | 0.976 |
|  | GPD | 56800.274 |  |
| 11 | LTB-scEDIII | 114607.224 | 1.123 |
|  | GPD | 102071.500 |  |

The densitometric comparision of expression levels was done using ImageJ 1.49v software [2].

References

1. Lim JG, Kim JA, Chung HJ, Kim TG, Kim JM, Lee KR, Park SM, Yang MS, Kim DH. Expression of functional pentameric heat-labile enterotoxin B subunit of *Escherichia coli* in *Saccharomyces cerevisiae*. J Microbiol Biotechnol. 2009;19:502–10.
2. Rasband WS, ImageJ, U. S. National Institutes of Health, Bethesda, Maryland, USA, https://imagej.nih.gov/ij/, 1997-2016.
